# *APOL1* variant-expressing endothelial cells exhibit autophagic dysfunction and mitochondrial stress

**DOI:** 10.3389/fgene.2022.769936

**Published:** 2022-09-27

**Authors:** Ashira Blazer, Yingzhi Qian, Martin Paul Schlegel, Huda Algasas, Jill P. Buyon, Ken Cadwell, Michael Cammer, Sean P. Heffron, Feng-Xia Liang, Shilpi Mehta-Lee, Timothy Niewold, Sara E. Rasmussen, Robert M. Clancy

**Affiliations:** ^1^ Division of Rheumatology, Department of Medicine, Hospital for Special Surgery, New York, NY, United States; ^2^ Division of Biostatistics, Department of Population Health, New York University School of Medicine, New York, NY, United States; ^3^ Division of Cardiology, Department of Medicine, New York University School of Medicine, New York, NY, United States; ^4^ Division of Rheumatology, Department of Medicine, New York University Grossman School of Medicine, New York, NY, United States; ^5^ Department of Microbiology, New York University Grossman School of Medicine, New York, NY, United States; ^6^ DART Microscopy Laboratory, New York University Grossman School of Medicine, New York University School of Medicine, New York, NY, United States; ^7^ Department of Obstetrics and Gynecology, New York University Grossman School of Medicine, New York, NY, United States

**Keywords:** *APOL1*, Autophagy, Mitochondria, HUVECs, interferon

## Abstract

Polymorphisms in the Apolipoprotein L1 (*APOL1*) gene are common in ancestrally African populations, and associate with kidney injury and cardiovascular disease. These risk variants (RV) provide an advantage in resisting *Trypanosoma brucei*, the causal agent of African trypanosomiasis, and are largely absent from non-African genomes. Clinical associations between the *APOL1* high risk genotype (HRG) and disease are stronger in those with comorbid infectious or immune disease. To understand the interaction between cytokine exposure and *APOL1* cytotoxicity, we established human umbilical vein endothelial cell (HUVEC) cultures representing each *APOL1* genotype. Untreated HUVECs were compared to IFNɣ-exposed; and *APOL1* expression, mitochondrial function, lysosome integrity, and autophagic flux were measured. IFNɣ increased median *APOL1* expression across all genotypes 22.1 (8.3 to 29.8) fold (p=0.02). Compared to zero risk variant-carrying HUVECs (0RV), HUVECs carrying 2 risk variant copies (2RV) showed both depressed baseline and maximum mitochondrial oxygen consumption (p<0.01), and impaired mitochondrial networking on MitoTracker assays. These cells also demonstrated a contracted lysosomal compartment, and an accumulation of autophagosomes suggesting a defect in autophagic flux. Upon blocking autophagy with non-selective lysosome inhibitor, hydroxychloroquine, autophagosome accumulation between 0RV HUVECs and untreated 2RV HUVECs was similar, implicating lysosomal dysfunction in the HRG-associated autophagy defect. Compared to 0RV and 2RV HUVECs, HUVECs carrying 1 risk variant copy (1RV) demonstrated intermediate mitochondrial respiration and autophagic flux phenotypes, which were exacerbated with IFNɣ exposure. Taken together, our data reveal that IFNɣ induces *APOL1* expression, and that each additional RV associates with mitochondrial dysfunction and autophagy inhibition. IFNɣ amplifies this phenotype even in 1RV HUVECs, representing the first description of *APOL1* pathobiology in variant heterozygous cell cultures.

## Introduction

Ancestrally African individuals, particularly those with autoimmunity, suffer from disproportionate rates of cardiovascular and kidney disease. Two polymorphisms, G1 (SER342GLY; ILE384MET) and G2 (6BP deletion N388/Y389), of the Apolipoprotein L1 (*APOL1*) gene have been shown to associate with these adverse phenotypes in individuals of recent African heritage. These polymorphisms have been evolutionarily conserved due to an advantage in resisting *Trypanosoma brucei*, the causal agent of African trypanosomiasis (Genovese et al., 2010), and are therefore largely absent from non-African genomes (Friedman et al., 2011; Ko et al., 2013). We previously found high allelic frequencies of the risk variants (RV) in an African American lupus cohort, where carrier status associated with progressive lupus nephritis and cardiovascular risk (Blazer et al., 2017). In large, population-based cohorts, these RVs associate with renal disease by multiple causes (OR: 1.5–2.0) (Parsa et al., 2013). Strikingly associations are much stronger in cohorts with chronic inflammatory or infectious conditions such as SLE (OR: 5.0–11.0) (Larsen et al., 2013; Blazer et al., 2021), HIV (OR: 30–80) (Kasembeli et al., 2015), and COVID-19 (Wu et al., 2020). Further exogenous interferon (IFN) exposure precipitates kidney injury in 2RV patients (Nichols et al., 2015). This phenomenon shows that inflammation enhances RV gene penetrance.

Consistent with its innate immune function, *APOL1* expression is highly responsive to inflammatory signals including toll-like receptor (TLR) ligation and inflammatory cytokines such as tumor necrosis factor (TNF) and interferon gamma (IFNɣ) (O'Toole et al., 2017). Immunoprecipitation assays show that interferon regulatory factor 1 and 2 and STAT2 bind the *APOL1* promoter heightening expression (Kent et al., 2002; Nichols et al., 2015). Therefore, *APOL1* RV penetrance may be contingent upon environmental “second hits” (Langefeld et al., 2018). Intracellular *APOL1* contains both a BH3 domain, which participates in initiating autophagy, and a pore-forming domain that can be inserted into phospholipid bilayers, causing tissue injury (Vanhollebeke and Pays, 2006; Wan et al., 2008). This injury is contingent upon *APOL1* protein accumulation beyond a toxic threshold. It is not currently clear whether heterozygous carriers may accumulate *APOL1* protein exceeding this threshold, or what conditions may cause this to occur (Blazer et al., 2019), and overexpression systems cannot answer that question. Recent clinical reports have found that even a single RV copy associates with advanced CKD in lupus nephritis (LN) (Vajgel et al., 2020).

Several cell types including podocytes, human embryonic kidney cells, and oocytes over-expressing variant *APOL1* have demonstrated mitochondrial injury, lysosome compromise, and autophagic flux defects resulting in cytotoxicity (Lan et al., 2014; Heneghan et al., 2015; Olabisi et al., 2016; Beckerman et al., 2017; Granado et al., 2017). However, these mechanisms have not been studied in vascular disease-relevant cell culture models (Ma et al., 2019). Moreover, the G1 and G2 SNPs reside in amino acid coding regions therefore altering protein structural stability and function (Sharma et al., 2016). Despite this apparent gain of function property (Bruggeman et al., 2019), the inheritance pattern is thought to be recessive and the literature has not described variant-associated injury in heterozygous cell cultures (Limou et al., 2014).

Endothelial dysfunction has been widely recognized as a risk factor for both vascular and kidney disease (Davignon and Ganz, 2004). Accordingly, this study was initiated to address the hypothesis that in HUVECs, variant *APOL1* confers mitochondrial stress, autophagy defects and loss of lysosome integrity. Cytokine exposure may additionally drive *APOL1* expression and amplify injury even in cells carrying a single RV. HUVECs were obtained from healthy controls of each genotype – 0RV, 1RV, and 2RV– to determine the consequences of cytokine exposure across *APOL1* genotype.

## Materials and methods

### Human subjects

This study was approved by the Institutional Review Board of New York University School of Medicine. HUVECs were obtained from healthy pregnant women from a single center labor and delivery ward. Participants provided written informed consent for fetal umbilical cord collection. Inclusion criteria were: African ancestry (concordant partner), and age >18 years. Umbilical cords that could not be processed within two hours of delivery were excluded. In total, 15 cords were collected. For experiments in which human sera were added to the HUVEC cultures (see below), serum samples were obtained from 5 SLE patients and 3 healthy controls who also provided informed consent. We have previously validated a functional assay to measure IFN activity in human serum samples (Niewold et al., 2007). IFN activity was measured in sera from SLE patients and healthy controls. These same samples were used as a pathological fluid and source of IFN to stimulate HUVEC *APOL1* expression. The SLE patients and healthy controls were African American and >18 years of age. Patients met at least 4 American College of Rheumatology (ACR) criteria for SLE (Hochberg, 1997). Clinical data at the time of sample draw included medications, ACR criteria, autoantibody profile, and SLE disease activity score.

### HUVEC culture establishment and processing

Please see the Major Resources Table in the online-only Supplementary Material for detailed descriptions of all antibodies and cultured cells used. A cut 5-cm section of umbilical cord was collected in RPMI media (Clonetics Corp.) supplemented with heparin 10 U/mL, penicillin/streptomycin 10 U/mL, and gentamycin 10 µg/mL. The umbilical vein was cannulated and perfused three times with HBSS solution to remove clotted blood. The umbilical vein was then perfused with collagenase A (type III) solution and both ends were clamped for 10 min to allow separation of umbilical vein cells as described (Crampton et al., 2007). Subsequently, the vein was re-cannulated and perfused again with HBSS, allowing cells to slowly drip from the vein into EGM-2 BulletKit Medium (Clonetics Corp.). The resulting solution was poured over a cell strainer. Cells were centrifuged and the pellet was re-suspended in clean culture media (EGM-2 supplemented with 10% FBS, 50 U/ml penicillin, 100 mg/ml gentamicin). The cell isolate contained HUVECs, fibroblasts, and residual blood cells. To yield enriched cultures of HUVECs, the cell suspension was passed through a magnetic bead column to capture CD146+ cells. The residual filtrate was discarded. HUVEC cultures were expanded and passaged for use in these experiments described below. Using FACS analysis, HUVECs exhibited strongly positive staining for both CD31 and CD146. In total, 15 healthy HUVEC cultures were established representing genotypes as follows: 0RV *n* = 8, 1RV *n* = 4, 2RV *n* = 3. There were no differences in donor infant sex distributions across genotype.

### *APOL1* genotyping

To ensure cell cultures representing each genotype in triplicate were available for subsequent analysis, *APOL1* genotyping was performed as described previously (Blazer et al., 2017). Briefly, genomic DNA was isolated from each HUVEC culture using the Qiagen kit (Valencia) according to the manufacturer’s instructions. DNA isolates were quantitated using a Nanodrop-1000 spectrophotometer (Nanodrop Products). One hundred ng of genomic DNA was used as a template for conventional polymerase chain reaction (PCR). A single 300 base-pair DNA segment containing the *APOL1* polymorphisms, G1 (rs73885319 and rs60910145) and G2 (rs71785313), was amplified using AmpliTaq Gold 360 DNA Polymerase (Applied Biosystems). For quality control, DNA was elongated in both forward and reverse directions using sequences 5’-GCC​AAT​CTC​AGC​TGA​AAG​CG-3’ and 5’-TGC​CAG​GCA​TAT​CTC​TCC​TGG-3’ respectively. Genotypes were analyzed using the GeneWiz online platform. Successful genotyping was completed on all DNA samples.

### Measurement of serum IFN-α activity

The reporter cell assay for IFN-α has been described in detail previously (Hua et al., 2006; Niewold et al., 2008). In this assay, reporter cells are used to measure the ability of patient sera to cause IFN-induced gene expression. The reporter cells (WISH cells, ATCC #CCL-25) are cultured with 50% patient serum for 6 h. The cells are lysed, cDNA is made from total cellular mRNA, and then quantified using real-time PCR. Forward and reverse primers for the genes: IFN-induced protein with tetratricopeptide repeats 1 (IFIT1), myxovirus resistance 1 (MX1), and dsRNA-activated protein kinase (PKR)--which are highly and specifically induced by IFN-α-- were used in the reaction (Niewold et al., 2008). The relative expression of these three genes in treated WISH cells was calculated as a fold increase compared to expression in WISH cells cultured with media alone. These values were standardized to healthy donors, and summed to generate a score reflecting the ability of sera to cause IFN-induced gene expression (serum IFNα activity) (Niewold et al., 2008). This assay has been highly informative in SLE and other autoimmune diseases (Harley et al., 2010; Niewold et al., 2011; Feng et al., 2012).

### Inflammatory model of *APOL1* expression

Single genotype HUVEC cultures grown in supplemented EGM-2 as above. Once confluent, cells were passaged and harvested once per week using trypsin-EDTA. Only HUVECs between passages 4–8 were used in experiments. For each genotype, untreated controls were compared to cells exposed to one of the following: 50% human sera isolated from healthy donors or patients with SLE, IFNɣ (50 pg/mL), IFNα (50 pg/mL) or TNF (50 pg/mL). Upon cell lysis, both protein and mRNA were extracted for immunoblot and qPCR.

### Immunoblot

Protein concentration was determined using a BCA protein assay kit (ThermoFisher Scientific) following manufacturer’s instructions. Appropriate concentrations of cell lysate were diluted with 4X Blot ® LDS sample buffer then heated to 70°C for 5 minutes. Samples were resolved on Blot 4-20% Bis-Tris Plus Gels (Life Technologies) and transferred to PVD membranes. Membranes were blocked with Odyssey ® Blocking Buffer (TBS) (Li-Cor Biotechnology) for 1 hour at room temperature. After blocking, membranes were incubated with rabbit anti-human *APOL1* (1 µg/mL) (Sigma-Aldrich) and mouse anti-human tubulin (1 µg/mL) (AbCam) diluted in 5% BSA/TBS-T overnight. Membranes were then incubated with an HCRP-conjugated anti-mouse or anti-rabbit secondary antibody (1:2000) (Santa Cruz Biotechnology) for 1 hour at room temperature. Protein bands were visualized using Li-Cor Image Studio Lite 4.0. Immunoblots were quantified by densitometry of experimental bands relative to loading controls using ImageJ 1.51 Java 1.8 running on Windows 7 or 10.

### Live cell imaging

HUVECs (1 × 10^4^ cells per 200 µL media; plate area 34 mm^2^) were seeded on Greiner Bio-One CELL view Cell Culture Slides (Fisher Scientific, Pittsburgh, PA) and allowed to adhere overnight. Cells were either left untreated in EGM-2 media, treated with 50 pg/mL of IFNɣ, or 50 pg/mL of IFNɣ plus 25 µM of non-selective lysosome blocker, hydroxychloroquine, in duplicate for each experiment (*n* = 4). Cells were then stained with LysoTracker red (LTR) probes (ThermoFischer Scientific, Waltham, Ma) and MitoTracker green (MTR) probes (ThermoFischer Scientific, Waltham, Ma) for 30 min. Media was replaced with serum free RPMI with glutamine (Mediatech Inc., Manassas, VA). When multiple slides were run, plates were staggered to prevent variation due to time elapsed since staining. Fluorescent microscopy was performed with a Nikon Eclipse Ti with a Plan Apo λ 60×/1.4 Oil Ph3 objective, narrow pass filters, and an Andor Zyla sCMOS 5.5 camera operated by Nikon Elements. For lysosome assessments, the fluorescence intensity as measured by the integrated density was scored using ImageJ 1.51 Java 1.8 running on Windows 7 or 10. For mitotracker images, mitochondrial network morphology per cell was assessed using the Mito-Morphology set of macros outfitted for the FIJI distribution of ImageJ as described (Valente et al., 2017). Data represented an average of 1000–1400 cells per genotype and condition, across five independent experiments. The tools and instructions for their usage can be found at https://github.com/ScienceToolkit/MiNA.

### Autophagy assessments

Autophagophore component proteins LC3-II/I were assessed by immunoblot. PVD membranes were treated with rabbit anti-LC3 primary antibodies (1 µg/mL) (Cell Signaling) diluted in 5% BSA/TBS-T followed by HCRP-conjugated anti-rabbit secondary antibodies (1:20000). Fluorescence units were quantified in the context of *APOL1* staining using ImageJ 1.51 Java 1.8 running on Windows 7 or 10.

In parallel, single HUVEC cultures of 40,000 cells per 300µL of cell media representing each genotype were plated on 0.1% gelatin-coated cover slips (BD Biosciences) housed in 24-well plates (1.9 cm^2^ per well). HUVECs were either left untreated or given 50pg/mL of IFNɣ. HUVECs representing each genotype were again given 50pg/mL of IFNɣ plus 25 µM of hydroxychloroquine. After exposure for 18 hr, HUVECs were washed with PBS and fixed with 3.7% formaldehyde for 10 minutes. The cover slips were again washed and cells permeabilized with 0.5% Triton for 20 minutes. Following an additional wash step, cover slips were treated with PBS gelatin solution as a blocking step for 1 hour. They were then stained with a DAPI DNA dye (Vector Laboratories) and primary antibodies to anti-human SQSTM1/p62 (Abcam) (both raised in rabbit) diluted in PBS gelatin solution at concentrations of 1:300 each. Cells were again washed and stained with anti-rabbit TRIT-C and Alexa-488 (Fisher Scientific) diluted in PBS gelatin at concentrations of 1:300. The cover slips were mounted on glass slides for visualization.

HUVECs were stained for p62 and the number of autophagic puncta (log transformed) per cell was observed. HCQ was again used to arrest the degradation of the autophagosomes by blocking lysosome acidification (Mauthe et al., 2018). Therefore, a comparison of *APOL1* genotype-dependent differences in autophagy at baseline, upon IFNɣ exposure and IFNɣ plus HCQ exposure (autophagic flux inhibition) was established. To assess the degree to which autophagic flux was impaired, the puncta count at baseline or upon IFNɣ exposure was compared to the HCQ condition. Fluorescent microscopy was performed with a Nikon Eclipse Ti as above. Images of cells were taken to make sure full cells were in the field for measurement. No choices were made based on morphology or intensity. All cells that were fully in each field were traced. A macroinstruction was written for ImageJ to locate discrete bright spots which were identified as puncta. A measurement including a 3-pixel-radius circle centered on each punctum was measured and, for each cell, summed. The integrated density of total puncta per cell was reported. Assessments were made by unmasked (AB) and repeated by a masked (HA) observer.

### Mitochondrial respirometry assay

Forty-thousand HUVECs representing each genotype (0RV, 1RV, and 2RV) were seeded on V7 cell culture plates (Seahorse Bioscience). Cells were either left untreated or exposed to IFNɣ (50 pg/mL) for 18 h. One hour prior to measurement, cell culture media was replaced with assay media (3 mM glucose, 1 mM sodium pyruvate, and 1.5 mM glutamine without FBS at a pH of 7.4). Port injections of oligomycin (1 μM), FCCP (0.25 μM) and rotenone/antimycin (1 μM each) were filled for bioenergetic profiling. Cellular respiration was measured using a Seahorse Bioscience XF 24-3 analyzer. This assay measures cellular mitochondrial function in real time using well-defined inhibitors, oligomycin, FCCP, and Antimycin A (Chacko et al., 2014; Son et al., 2017). Baseline oxygen consumption rate (OCR) was first measured, followed by the addition of the ATPase inhibitor, oligomycin, in order to evaluate the non-ATPase-dependent OCR. These measurements were used to assess overall BHI, which is proportional to reserve capacity and ATP synthase-dependent OCR and inversely proportional to proton leak and non-mitochondrial OCR (Chacko et al., 2014).

### Electron microscopy

Cultured cells were fixed in 2.5% glutaraldehyde and 2% paraformaldehyde in 0.1M sodium cacodylate buffer (pH 7.2) for 2 h at 4^o^C and post-fixed with 1% osmium tetroxide for 1.5 h at room temperature, then processed in a standard manner and embedded in EMbed 812 (Electron Microscopy Sciences, Hatfield, PA). Ultrathin sections (60 nm) were cut, mounted on copper grids and stained with uranyl acetate and lead citrate by standard methods. Stained grids were examined under a Talos120C electron microscope and photographed with a Gatan OneView camera. Twenty random cells in each sample were imaged for morphological analysis. Assessments were made by unmasked (AB) and repeated by masked (HA, FXL) observers.

### Statistical analysis

For each genotype and experimental condition, non-normally distributed data were expressed as median and interquartile range, and normal data were expressed as mean ± standard deviation. Medians were used when the population could not be assumed to be normally distributed. Where observed sample sizes were larger and data were normally distributed, ANOVA for multiple-group comparisons were used. Data normality was assessed by visual examination of the observed distributions and Kolmogorov-Smirnov tests. Equality of variance was assessed by F-tests. Integrated densities were log-transformed to better satisfy normality in the p62 staining experiments. If F-tests failed to reject the hypothesis of equal variances, two sample t-tests with equal variances for two-group comparisons and ANOVA for multiple-group comparisons were used instead. When ANOVA tests rejected the null hypothesis, post hoc pairwise comparisons were performed. All statistics were carried out using IBM SPSS or R v4.6 software. The level of significance was set at 0.05.

## Results

### Inflammatory stimuli increase HUVEC *APOL1* expression

**FIGURE 1:**
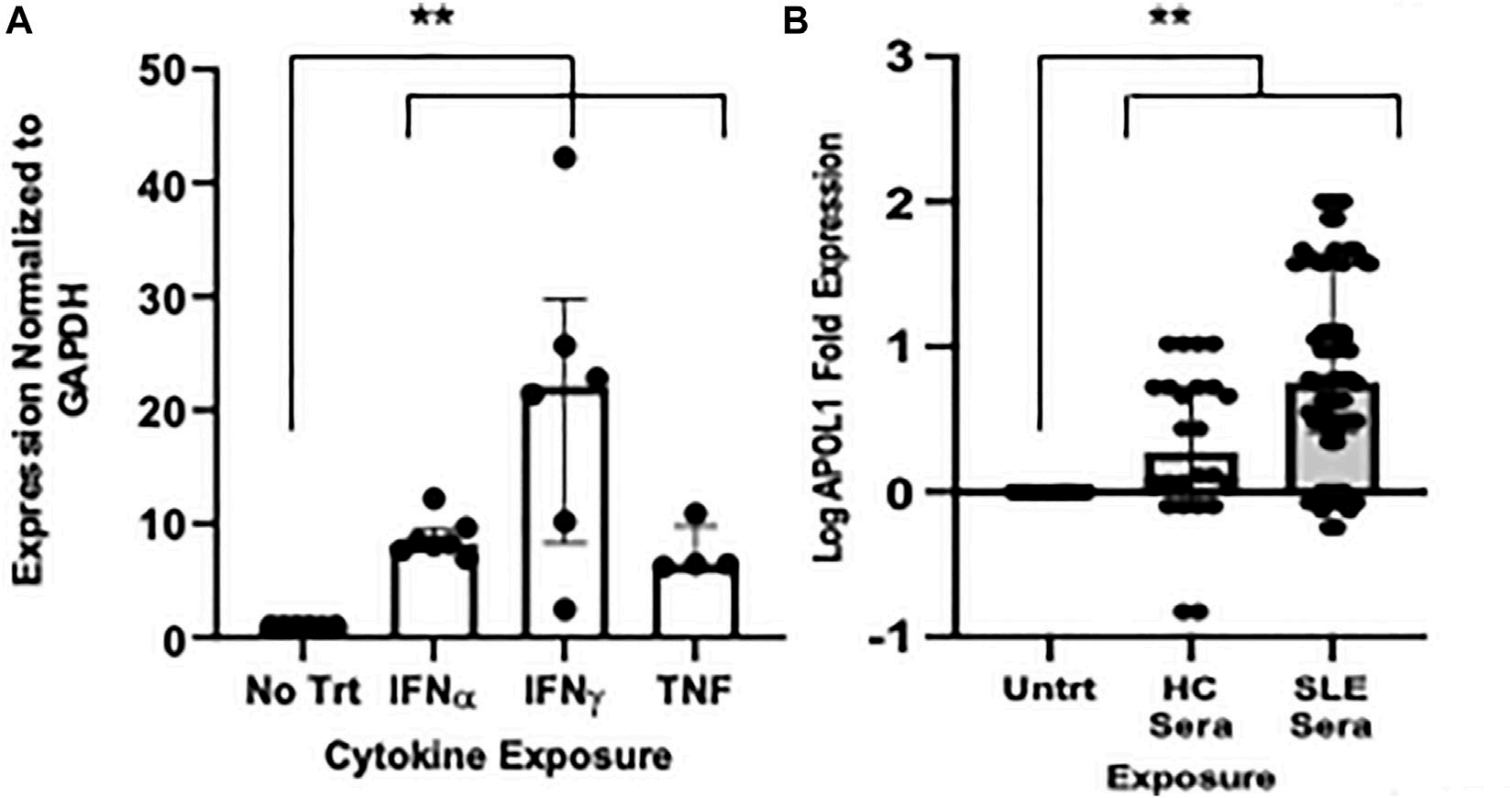
Endothelial cells treated with inflammatory cytokines induce *APOL1* expression. **(A)** *APOL1* expression in untreated HUVECs compared to IFNα (50 pg/ml), IFNɣ (50 pg/ml), and TNF (10 ng) exposed for 18 h (average of 5 experiments, 9 HUVEC donors). Shown on the *y*-axis are 2^−ΔΔCT^ (transcript normalized to GAPDH) values and shown on the *x*-axis are cytokine exposures. **(B)** Exposure of HUVECs to sera at 1:1 dilution for 18 h resulted in an upregulation of *APOL1* transcription (average of 5 experiments, 9 HUVEC donors). Shown on the *y*-axis are log 2^−ΔΔCT^ (transcript normalized to GAPDH) values and shown on the *x*-axis are exposure conditions. Comparisons are made between the median fold expression in untreated vs. exposure conditions using Kruskal–Wallis tests (both 1A and 1B *p* < 0.02 as indicated by **) followed by post hoc Dunn test. Abbreviations: Untrt = untreated condition, IFNα = interferon alpha, IFNγ = interferon gamma, TNF = tumor necrosis factor, HC Sera = healthy control sera, and Systemic Lupus Erythematosus Sera = SLE Sera, experiments were run in triplicate.

For each experiment, the number of individual HUVEC donors is outlined in Supplementary Figure S1. The capacity of SLE-associated cytokines including IFNα, IFNɣ, and TNF to stimulate gene expression in HUVECs was tested. Exposing HUVECs to IFNα, IFNɣ, and TNF resulted in increased *APOL1* expression at a median and IQR of 8.3 (7.7 to 9.6), 22.1 (8.3 to 29.8), and 6.4 (6.3 to 9.8) fold respectively, versus untreated HUVECs (for each cytokine vs untreated, p<0.02) (Figure 1A). Given that IFNɣ most robustly induced *APOL1* expression, this cytokine was carried forward for all subsequent experiments. On immunoblot, the median IFNɣ-exposed HUVEC *APOL1* protein expression was 8.02 (3.4 to 27.9)-fold that of untreated HUVECs (*p* = 0.001; Supplementary Figure S1G shows a representative immunoblot). Sera from SLE patients (N = 5) and controls were incubated with HUVECs across genotypes, and *APOL1* expression was assessed. In response to SLE sera, the median *APOL1* expression increased 6.0 (2.8 to 39.8)-fold compared to 2.0 (0.9 to 5.3)-fold in healthy control sera (SLE sera-treated vs. HC sera treated, *p* = 0.01). Log-fold change is shown in Figure 1B; clinical data for sera donors are shown in Table 1. This increased expression was apparent across genotype (Supplementary Figures S1A–F). Upon genotyping the PCR products, we found that genotypes were concordant with chromosomal DNA and 1RV HUVECs expressed both variant and ancestral *APOL1* alleles.

**TABLE 1:** African American (0RV genotype) SLE Sera Donor Demographics, SLE Activity, and IFN score.

Subject							
	SLE 1	SLE 2	SLE 3	SLE 4	SLE 5	HC1	HC2
Demographics							
Age (years)	62	33	29	47	31	34	26
Sex	F	F	F	F	F	F	F
SLE Activity							
dsDNA	1	927	181	12	76	NA	NA
C3 (mg/dl)	91	49	68	100	79	NA	NA
C4 (mg/dl)	16	12	7	15	9	NA	NA
IFN Score (units)	28.2	219.7	167.6	1.1	876	1.9	0.8

Demographics and clinical characteristics of SLE and healthy control (HC) serum donor subjects at the time of blood draw. All subjects were African American and *APOL1* ancestral allele homozygous.

### *APOL1* variant carrying HUVECs exhibit defects in mitochondrial respiration

**FIGURE 2:**
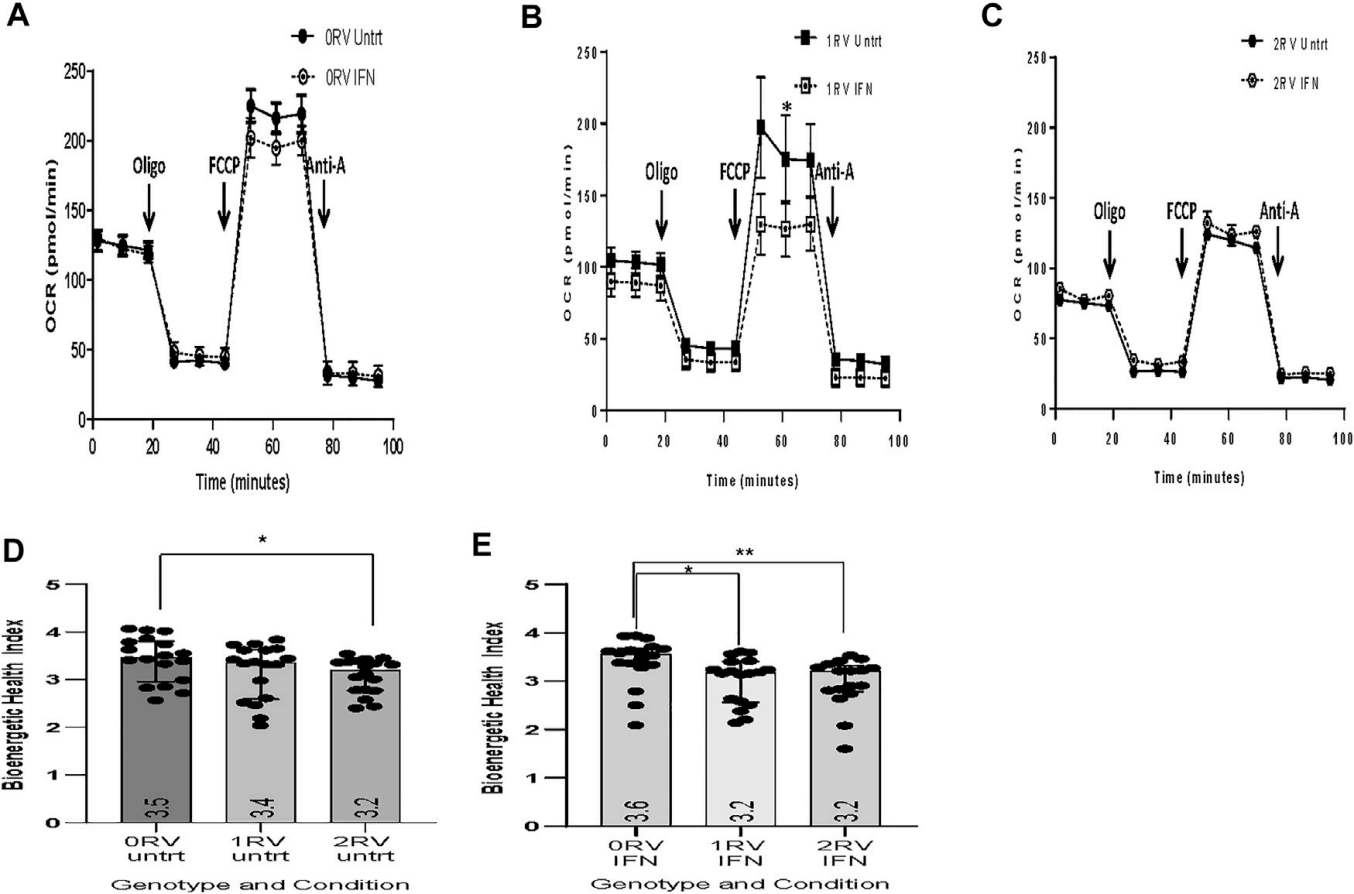
The bioenergetic profiles of HUVECs across genotype. The bioenergetic profiles of HUVECs across genotype. *APOL1* risk variant associations with attenuated mitochondrial function including maximum respiration, reserve respiration capacity, and bioenergetic health index. Live HUVEC metabolic assays were performed using the Seahorse XF platform. Representative tracings by *APOL1* genotype are shown in figures **(A**–**C)**. In this assay, oxygen consumption rate (OCR) was measured at baseline, upon oligomycin (oligo), FCCP, and antimycin A exposure. **(D**,**E)** Bioenergetic health index (BHI) calculated by *APOL1* genotype and exposure (5 experiments averaged representing 9 HUVEC donors). Genotypes are represented as follows: 0RV, 1RV, and 2RV (RV = risk variant). Exposure conditions included: no treatment (Untrt) or IFNƔ (50 pg/ml, 18 h). *p*-values were calculated using Kruskal–Wallis (KW) one-way analysis of variance for cross genotype comparisons. Where the KW models were significant, post-hoc Dunn’s multiple comparisons tests were completed. * indicates *p* < 0.05, and ** indicates *p* < 0.01.

We measured oxygen consumption rate (OCR) in HUVECs of each genotype by exposure (observations: *n* = 108). Baseline, OCR was higher in 0RV compared to 1 or 2RV HUVECs with mean and SD of (89.9±5.6 pmol/min vs 71.7±4.5 pmol/min vs 66.5±3.2 pmol/min respectively; *p* = 0.002). Likewise, maximum OCR was higher in 0RV HUVECs; values dropped with each RV copy, with means of 152.7±10.7 pmol/min in 0RV, 122.3 ± 9.6 pmol/min in 1RV, and 102.6±4.6 pmol/min in 2RV HUVECs (*p* = 0.001). With the addition of IFNɣ, mean maximum OCR fell to 133.2 ± 11.3 pmol/min, 100.8 ± 6.2 pmol/min, and 92.9 ± 6.1 pmol/min in the 0, 1, and 2 RV HUVECs respectively (*p* = 0.002). Figures 2A–C shows representative OCR curves across the genotypes. Differences in mitochondrial respiration did not result in statistically significant differences in apoptosis or cell death (Supplementary Figures S2A–E).

As shown in Supplementary Figures S2D,E, the median bioenergetic health index (BHI) was significantly lower in the untreated 2RV HUVECs compared to the 0RV or 1RV HUVECs (0 RV = 3.5 (2.9 to 3.8), 1 RV = 3.4 (2.6 to 3.6), 2 RV = 3.2 (2.8 to 3.4); *p* = 0.04). Exposing HUVECs to IFNɣ further decreased BHI in 1RV HUVECs resulting in significant differences between 0RV compared to 1RV and 2RV (median BHI: 0RV = 3.6 (3.3 to 3.7), 1RV = 3.2 (2.6 to 3.4), 2RV = 3.2 (2.8 to 3.3); *p* = 0.003). Results by experiment and sample are shown in Supplementary Figure S3. While the mitochondrial phenotype was apparent in both treated and untreated 2RV HUVECs, 1RV HUVECs exhibited a difference only after IFNɣ exposure, again suggesting an inducible phenotype.

### Mitochondrial structure

**FIGURE 3:**
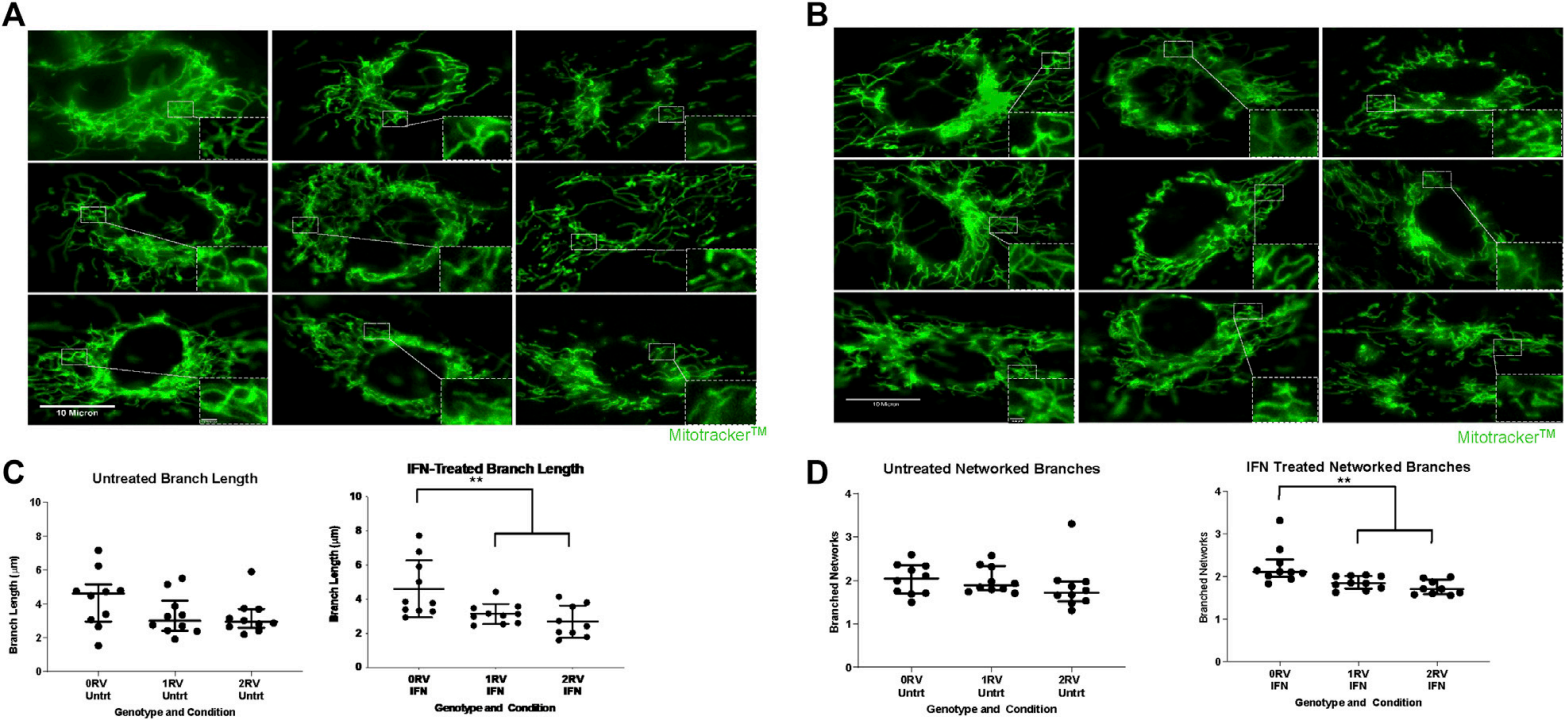
Assessment of mitochondrial structure in resting and stimulated HUVECs using MitoTracker, a fluorescent proxy of ultrastructure. Assessment of mitochondrial structure in resting and stimulated HUVECs using MitoTracker fluorescent mitochondria stain. **(A**,**B)** Mitochondrial structure including branch length and networking are shown in HUVECs representing each genotype, 0 risk variant (0RV left columns), 1 risk variant (1RV middle columns), and 2 risk variants (2RV right columns) were either left untreated **(A)** or exposed to IFNɣ 50 pg/ml **(B)** for 18 h. Representative immunofluorescence (MitoTracker green stained) images are shown. Ten cells per genotype and condition were measured; in total aggregate data from 12 HUVEC donors and 4 experiments were analyzed. **(C**,**D)** Quantified mitochondrial branch length **(C)** and networked branches **(D)** by *APOL1* genotype and exposure condition (*x* axis) using the Mitochondrial Network Analysis (MiNA) tools available in the FIJI distribution of ImageJ. Mitochondrial length was measured in microns (μM). Each additional risk variant associated with a reduced degree of mitochondrial networking; and effect that became statistically significant across the genotypes upon treatment with IFNɣ. *p*-values were calculated using Kruskal–Wallis test for cross genotype comparisons. *p* < 0.01 is indicated by **.

HUVECs of each genotype were either left untreated or given IFNɣ overnight. Subsequently, mitochondrial ultrastructure was evaluated by fluorescent microscopy and transmission electron microscopy (TEM). For the former, sets of 10 HUVEC images across genotypes and exposure conditions were evaluated for mitochondrial morphology per cell including length and number of branches using the MiNA workflow in ImageJ. This workflow processes fluorescent images by enhancing contrast, creating a sharp mask, and creating a skeletonized image amenable to length and networking measurements (Valente et al., 2017) (representative images shown in Figures 3A,B). In untreated HUVECs, the median distribution of branch length and networking across the genotypes were 0RV: 4.5 μm (2.9 to 5.1 μm) 1RV: 3.0 μm (2.4 to 4.2 μm) 2RV: 2.9 μm 2.6 to 3.6 μm; p= 0.2 and 0RV: 2.0 (1.7 to 2.3), 1RV: 1.9 (1.8 to 2.3), and 2RV: 1.7 (1.5 to 1.9) respectively (Figures 3C,D). Upon exposure to IFNɣ , genotype-associated differences became significant (median branch length: 0RV: 3.8 μm (3.3 to 6.2 μm), 1RV: 3.1 μm (2.6 to 3.5 μm) 2RV: 2.4 μm (1.9 to 3.7 μm); p=0.01. Median networked branches were significantly lower in 1 and 2RV HUVECs as follows: 0RV: 2.1 (2.0 to 2.4) 1RV: 1.8 (1.7 to 2.0) 2RV: 1.7 (1.6 to 1.9); *p* = 0.001. Post hoc 2 group comparison 0RV vs 1RV *p* = 0.04; 0RV vs. 2RV *p* = 0.0005; Figures 3C,D). TEM images were concordant with this finding. Untreated HUVECs across genotype showed small differences in mitochondrial area (median area for 0, 1, and 2 allele HUVECs=0.09 μm^2^, 0.08 μm^2^, 0.07 μm^2^ respectively) (Supplementary Figure S4). Upon IFNɣ exposure, these differences became more pronounced (median area + IFNɣ 0 RV=0.11 μm^2^; 1 RV=0.10 μm^2^; 2 RV=0.07 μm^2^ p <0.001) (Supplementary Figures S4A,B).

### *APOL1* variant-Carrying HUVECs exhibit lysosomal defects

**FIGURE 4:**
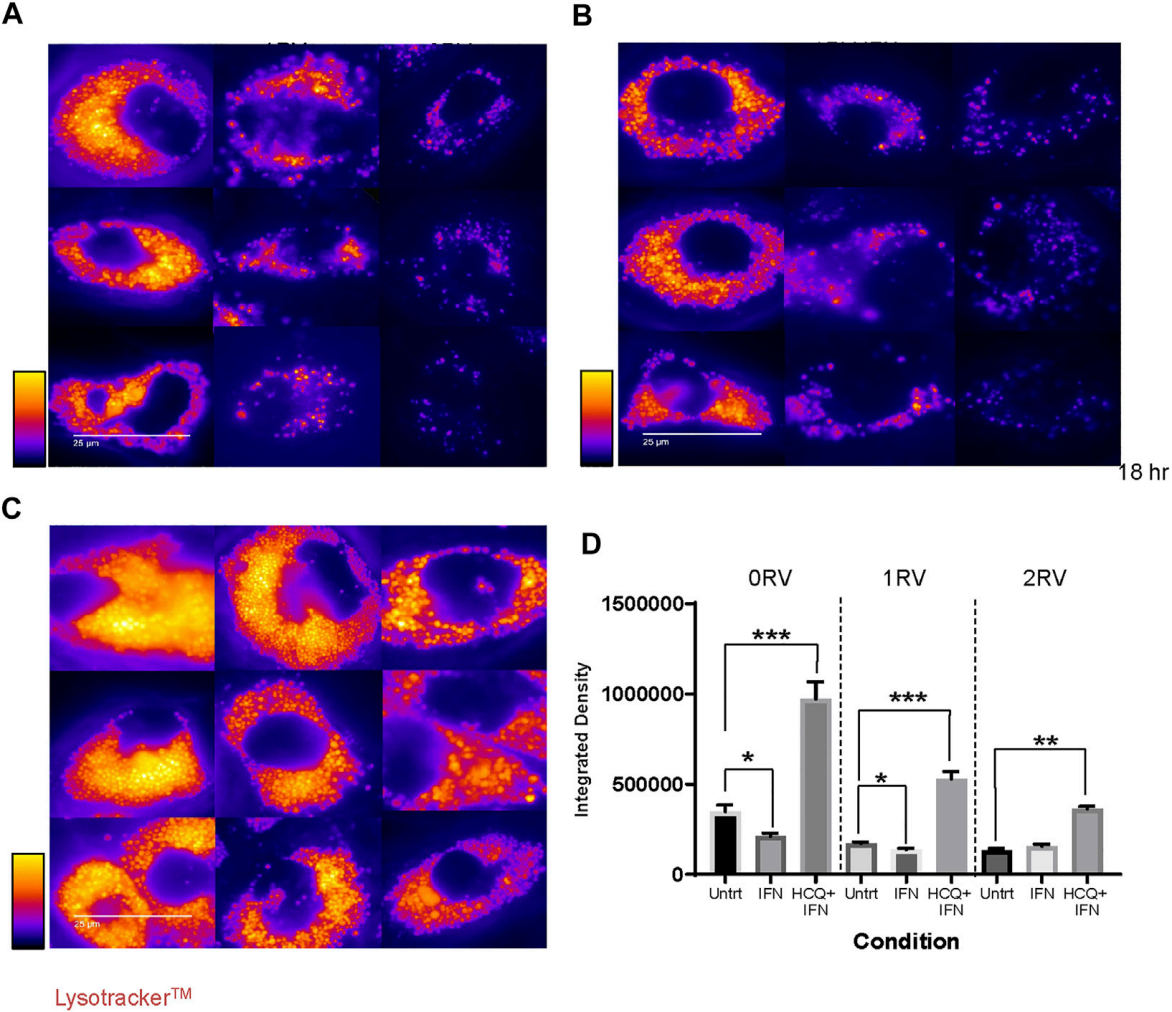
Assessment of lysosomal structure in resting and stimulated HUVECs with or without hydroxychloroquine (HCQ) using LysoTracker. Assessment of lysosomal structure in resting and IFNɣ-exposed or hydroxychloroquine plus IFNɣ-exposed HUVECs using LysoTracker, a fluorescent proxy for lysosomes. Fluorescent micrographs were converted to single channel gray scale, and pseudocolor images representing staining intensity are shown on a continuous scale as depicted by the heat map keys to the left of the images. **(A)** Immunofluorescent images of untreated HUVECs representing each genotype, 0RV (left column), 1RV, and 2RV (right column). **(B)** Representative images of IFNɣ 50pg/ml-treated HUVECs across genotype (parallel layout as per A) for 18 h overnight. **(C)** Representative images of HCQ (25 µM) plus IFNɣ-treated HUVECs across genotype (layout as per A) LysoTracker staining was performed. Images were captured by florescent microscopy using a Nikon Eclipse Ti and representative microphotographs were selected. **(D)** The average lysosome intensity per cell region of interest (Integrated Density) for each genotype and treatment condition group. N = 1,000–1,400 cells per genotype and condition. *p*-values were calculated using Kruskal–Wallis test for cross genotype comparisons, and Wilcoxon rank sum test for comparing untreated group to the IFNγ treated or HCQ plus IFNγ group.*p < 0.05; **p < 0.01, ***p < 0.001.

At baseline, 2 RV HUVECs exhibited significantly lower lysotracker staining intensity than 0 or 1 RV HUVECs (p<0.001) (Figure 4A; over 1,500 observations per genotype. IFNɣ exposure significantly decreased lysotracker staining intensity in the 0 and 1 RV HUVECs (*p* = 0.04 and *p* < 0.001 respectively). In 2RV HUVECs, lysosome staining intensity did not change with IFNɣ exposure but remained lower than that of the other two genotypes (Figure 4B). HUVECs were exposed to both IFNɣ and HCQ. HCQ is a reagent that blocks lysosome acidification thereby arresting both organelle function and turnover (Steinman et al., 1983; Klionsky et al., 2016). Exposing HUVECS to 25 µM hydroxychloroquine (HCQ) did not influence HUVEC *APOL1* expression (Supplementary Figure S1G). In all conditions, the co-exposure of IFNɣ and HCQ resulted in increased lysosome staining intensity (Figure 4C). This effect, however, was significantly less in both 1 and 2 RV-carrying cells. Lysosome staining intensity is quantified in Figure 4D, and results by experiment and sample are shown in Supplementary Figures S6A–E shows a low-power representative image of HUVECs across genotype and treatment condition. Taken together, each additional RV copy associated with less HUVEC lysosome staining intensity—a phenotype that became more pronounced with IFNɣ exposure.

### *APOL1* variant-carrying HUVECs display defects in autophagic flux

**FIGURE 5:**
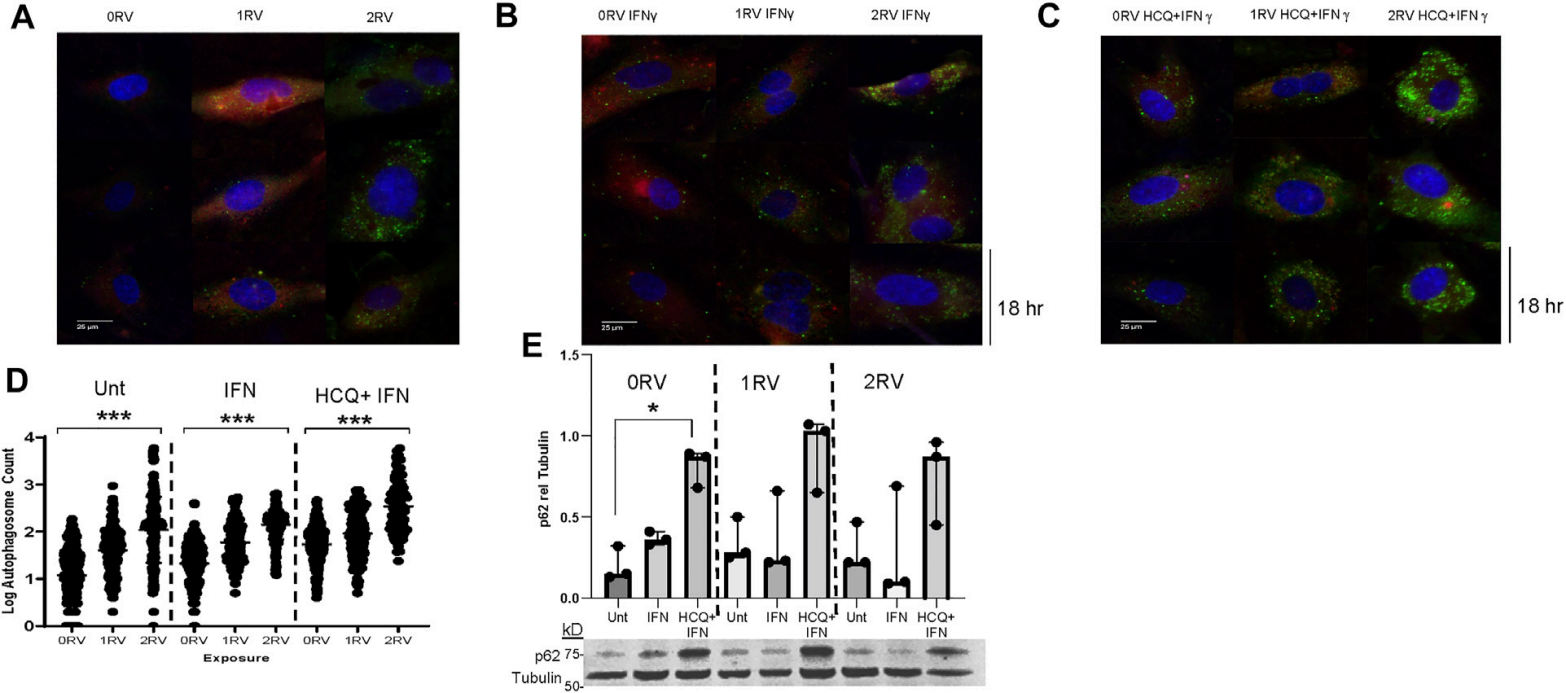
*APOL1* risk variant-carrying HUVECs display autophagic flux deficiencies. *APOL1* risk variant-carrying HUVECs display autophagic flux deficiencies. Assessment of autophagosome accumulation using SQSTM1 (p62) staining, a proxy for autophagic flux inhibition. **(A)** Representative immunofluorescence images of untreated HUVECs stained for SQSTM1 (p62) across *APOL1* genotype. Green FITC positive puncta represent p62-positive autophagosomes. Tubulin counterstain is represented in red: 0 risk variants (RV) (left column), 1RV (middle column), 2RV (right column). **(B)** Representative immunofluorescence images of IFNɣ (50 pg/ml)-exposed HUVECs **(C)** Representative immunofluorescence images of IFNɣ plus HCQ (25 µM)-exposed HUVECs. **(D)** Log transformed values of p62 positive puncta per cell (*y* axis) and the exposure condition (*x* axis) are shown. HUVEC genotype is labeled from left to right. **(E)** Immunoblot of HUVEC lysates showing p62 protein concentration compared to tubulin loading control by genotype and exposure condition. Quantification of experiments are done in triplicate (median + IQR) shown above. HUVECs were treated for 18 h. *p* values were calculated from Kruskal–Wallis one-way analysis of variance for cross genotype comparisons. Where models rejected the null hypothesis, a post-hoc 2 group analysis was performed. * represents *p* value < 0.05, ** represents *p* value <0.01, *** represents *p* value < 0.001.

Autophagosome maturation and degradation (flux) is contingent upon a functioning lysosome (Liu et al., 2016) which was demonstrated to be compromised in *APOL1* variant-carrying HUVECs (above). Therefore, autophagosomes were evaluated using both fluorescent microscopy of autophagophore shuttle protein, SQSTM1 (p62), and TEM. HUVECs were stained for p62 and the number of autophagic puncta (log transformed) per cell was observed. In untreated HUVECs, autophagosome count was the lowest in 0 RV cells and increased with each additional RV (log autophagosome count per genotype: 0RV: 1.1 ± 0.57; 1RV: 1.6 ± 0.48; 2RV: 2.0 ± 0.70, p<0.001. Representative images are shown in Figure 5A). Across genotypes, IFNɣ exposure increased autophagosome count (0 RV: 1.3 ± 0.45, 1 RV: 1.8 ± 0.44, 2RV: 2.2 ± 0.31, *p* < 0.001 representative images shown in Figure 5B). In each genotype group, the number of autophagosomes increased in the IFNɣ plus HCQ–treated condition; however, 2RV HUVECs exhibited the highest autophagosome count (0 RV: 1.7 ± 0.38, 1 RV: 2.0 ± 0.5, 2 RV: 2.5 ± 0.55, *p* < 0.001 representative images shown in Figure 5C). Autophagosomes were confirmed on TEM by genotype and exposure condition (Supplementary Figure S8). Microscopy results are quantified in Figure 5D, and confirmed by p62 immunoblot in Figure 5E. Results by experiment are shown in Supplementary Figure S7. A supporting immunoblot of the LC3 II/I ratio by genotype and exposure condition is shown in Supplementary Figure S9. Taken together, these results support an association between HUVEC *APOL1* genotype and autophagic flux inhibition. Congruent with the lysosome staining results, each additional RV had an effect on autophagic flux with IFN exacerbating the phenotype–particularly in the heterozygous condition.

## Discussion

We demonstrate that the African ancestry *APOL1* RVs have a number of adverse cellular impacts in endothelial cells, which are exacerbated by IFNγ. This is demonstrated in a system in which *APOL1* is regulated under physiological conditions and present in the observed natural allelic copy numbers. Our results are congruent with recent literature reports that *APOL1* RVs associate with functional consequences such as decreased mitochondrial metabolic potential and mitochondrial fragmentation (Ma et al., 2020). Specifically, in 2RV and IFNɣ-treated RV heterozygous HUVECs, maximum respiration, reserve respiration capacity, glycolytic capacity, and bioenergetic health index were attenuated relative to the ancestral allele homozygous HUVECs. Using a parallel set of conditions, the presence of 1RV or 2RVs also associated with decreased lysosome staining by lysotracker as well as an inhibition of autophagocytic flux, which was based on fluorescent evaluations of p62 and confirmed by TEM. Taken together, these data support that 2 RVs—and under inflammatory stress 1 RV—contribute to a senescent endothelial cell phenotype, which is characterized by overall lower energy production and untoward consequences to autophagosome maturation and degradation (flux).

*APOL1* high risk genotype (HRG), defined as two variants in any combination (G1/G1, G1/G2, or G2/G2), has been associated with several adverse phenotypes including chronic kidney disease and microvascular stroke; however, these associations are highly variable. For example, population based studies show no appreciable effect of HRG on the development of diabetic nephropathy (Freedman et al., 2018). The odds of developing chronic kidney disease range from 1.5–2.0 in general cohorts, to 2.5–7.3 in SLE nephritis, to 29–80 in HIV-associated nephropathy (Kopp et al., 2011; Larsen et al., 2013; Parsa et al., 2013). A similar trend is emerging in vascular disease. Upon stratifying by diabetes status, Gutierrez et al. found null associations in diabetics, but hazard ratios of 5.1 for small vessel strokes in non-diabetics. (Gutierrez et al., 2018) Inflammatory conditions, such as SLE may be an important context under which HRG confers risk. HRG has been shown to associate with SLE collapsing glomerulopathy and progression to end-stage kidney disease (Larsen et al., 2013; Freedman et al., 2014).

The *APOL1* gene product may cause mitochondrial dysfunction by several mechanisms. *APOL1* is a five-domain protein with several intracellular functions (O'Toole et al., 2017; Heneghan et al., 2015; Sharma et al., 2016; Blazer and Clancy, 2017). Its expression is up-regulated by cellular stress including inflammatory signals, nutrient deprivation, and hypoxia (Vanhollebeke and Pays, 2006; Wan et al., 2008). *APOL1*’s colicin-like pore forming domain may be inserted into cell membrane, lysosome, or mitochondrial phospholipid bilayers in a pH dependent fashion (Thomson and Finkelstein, 2015). The G1 and G2 mutations allow for pore formation at lower levels of *APOL1* gene expression (Thomson and Finkelstein, 2015). Mitochondria may be injured by *APOL1* variants either directly, or indirectly due to defects in lysosomes and autophagic flux. *APOL1* has been shown to cause toxicity by disrupting lysosomes in human podocytes (Lan et al., 2014). Lysosome membrane permeability allows escape of lysosome hydrolases, including cathepsin-B, which mediate mitochondrial outer membrane permeability and loss of inner-membrane potential (Kroemer and Jaattela, 2005). Finally, others have shown that the *APOL1* pore co-localizes with the mitochondria in human embryonic kidney cells, directly causing membrane permeability (Ma et al., 2017). Future studies exploring these mechanisms in *APOL1* risk variant-carrying tissues could better explain additional features of RV pathobiology.

While there are studies of *APOL1* in cell culture, most have employed overexpression systems to deliver the gene (Lan et al., 2015; Nichols et al., 2015; Olabisi et al., 2016). These models showed that *APOL1* variant expression coincides with mitochondrial fragmentation, lysosome compromise, and an abundance of autophagosomes (Lan et al., 2014; Granado et al., 2017). However, exaggerated *APOL1* expression beyond that expected in native cells poses a limitation on the clinical interpretation of these results. Moreover, the viral transduction of cells could induce *APOL1* expression, autophagy, or other injury pathways, making it more difficult to assign risk variant-mediated effects (Kupin, 2017). This study, however, is not without limitations. Though experiments were performed across multiple donors of each genotype, the possibility that non-*APOL1*-related genetic variation contributed to our findings cannot be excluded. Though we included HUVECs of each genotype in triplicate, our limited sample size did not allow us to identify differences in phenotype across the combinations of G1 or G2 variants within the 2RV cells. Both male and female samples were utilized, and despite even distribution across genotype, differences in autophagy due to sex cannot be excluded. We chose p62 as an indicator of autophagic flux, however this protein has been shown to participate in other processes such as cellular stress and antioxidant response (Acar et al., 2021). Also, HUVEC cells may not recapitulate endothelial cell behavior in other vascular beds more relevant to atherosclerotic and kidney disease. Finally, the threshold at which *APOL1* expression influences lysosome function is not clear based on this study. Further work determining the intracellular concentration of *APOL1* and timing of lysosome injury is warranted given the above findings.

In sum, variant *APOL1* expression, particularly in the presence of inflammatory stimuli, confers endothelial cell dysfunction manifested as mitochondrial stress, lysosomal dysfunction and impaired autophagic flux. Exposures aimed at compensating for these metabolically compromised cellular states may improve the vascular consequences facing *APOL1* variant carriers, and may be particularly important in those people with inflammatory, infectious, or autoimmune diseases.

## Data Availability

The raw data supporting the conclusions of this article will be made available by the authors, without undue reservation.
