# Supplementary material for: APOL1 variant-expressing endothelial cells exhibit autophagic dysfunction and mitochondrial stress
Source: Front Genet. 2022 Sep 27;13:769936. doi: 10.3389/fgene.2022.769936 (PMC9551299; doi:10.3389/fgene.2022.769936)

Supplemental Figure Set 1. APOL1 expression by treatment conditions and varied genotypes

Quantification of APOL1 mRNA expression by qPCR in HUVECs across genotype (data separated by HUVEC genotype, n=12 HUVEC donors). **A-C.** Compared to untreated HUVECs, cells treated with IFNα (50pg/mL), IFNɣ (50pg/mL), and TNFα (10ng) for 18 hours show increased APOL1 expression across genotypes**. D-F.** Compared to untreated and healthy control (HC) sera treated cells, both IFNɣ and SLE sera-treated cells (average of 5 SLE sera responses represented) significantly increased APOL1 expression regardless of genotype.

A.

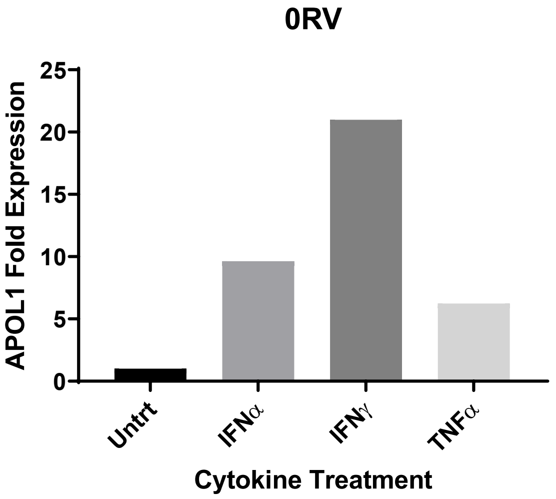

B.

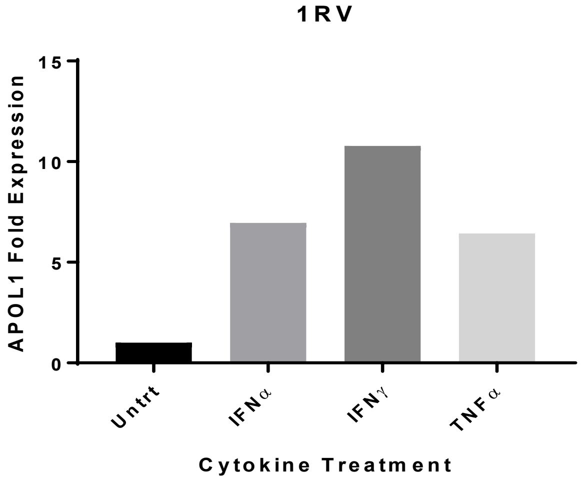

C.

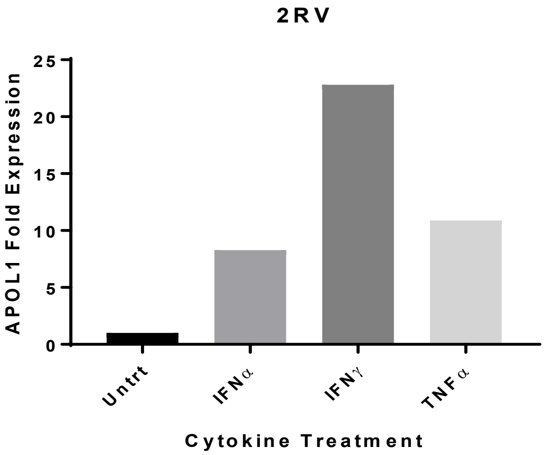

D.

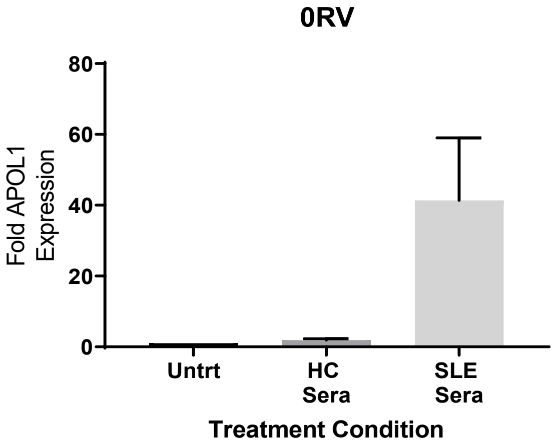

E.

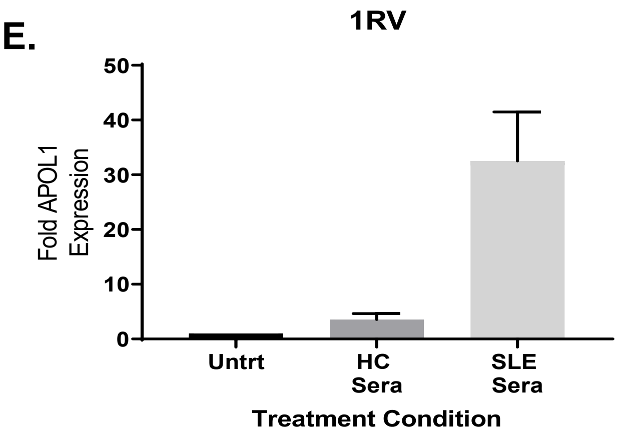

F.

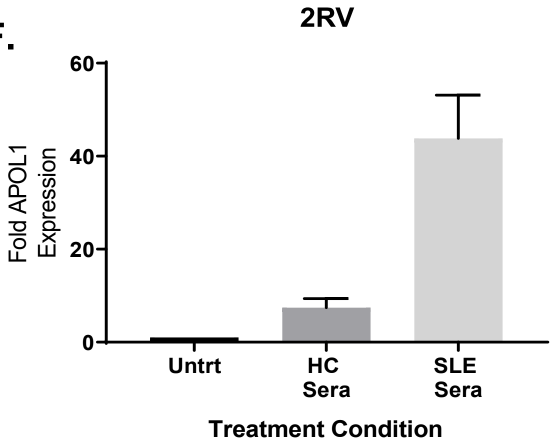

G.

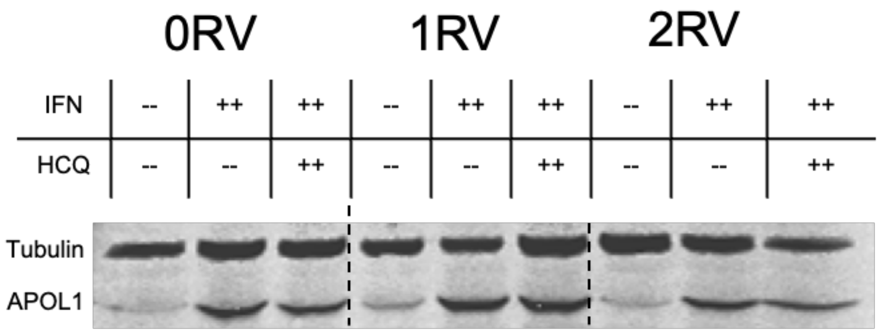

Supplemental Figure Set 2: Flow Cytometry for Apoptosis/necrotic Events by APOL1 Genotype and Condition: A. Primary HUVECs were stained with Annexin V/PI and representative flow cytometry scatter plots are shown. **B-D.** Quantification of flow cytometry data (n=3 experiments)**.** There were no statistically significant differences in apoptosis or necrosis across genotype or treatment condition as measured by Annexin V (Anx V) and Propidium Iodide (PI) staining**. E.** LDH Release Assay was completed to examine variability in cytotoxicity across genotype and treatment condition. The data revealed main between-subjects effect for genotype (F= 2.9 p= 0.07) that trended toward significance with 2RV HUVECs secreting more LDH. There was also a main within subjects effect for IFN treatment with less LDH release in IFN treated cells (F= 0.596, p=0.047). Finally there was a significant genotype by treatment interaction F=0.092, p=0.015) with a larger decrease in LDH release upon IFN treatment in 2RV carrying cells compared to 0 or 1RV carrying cells.

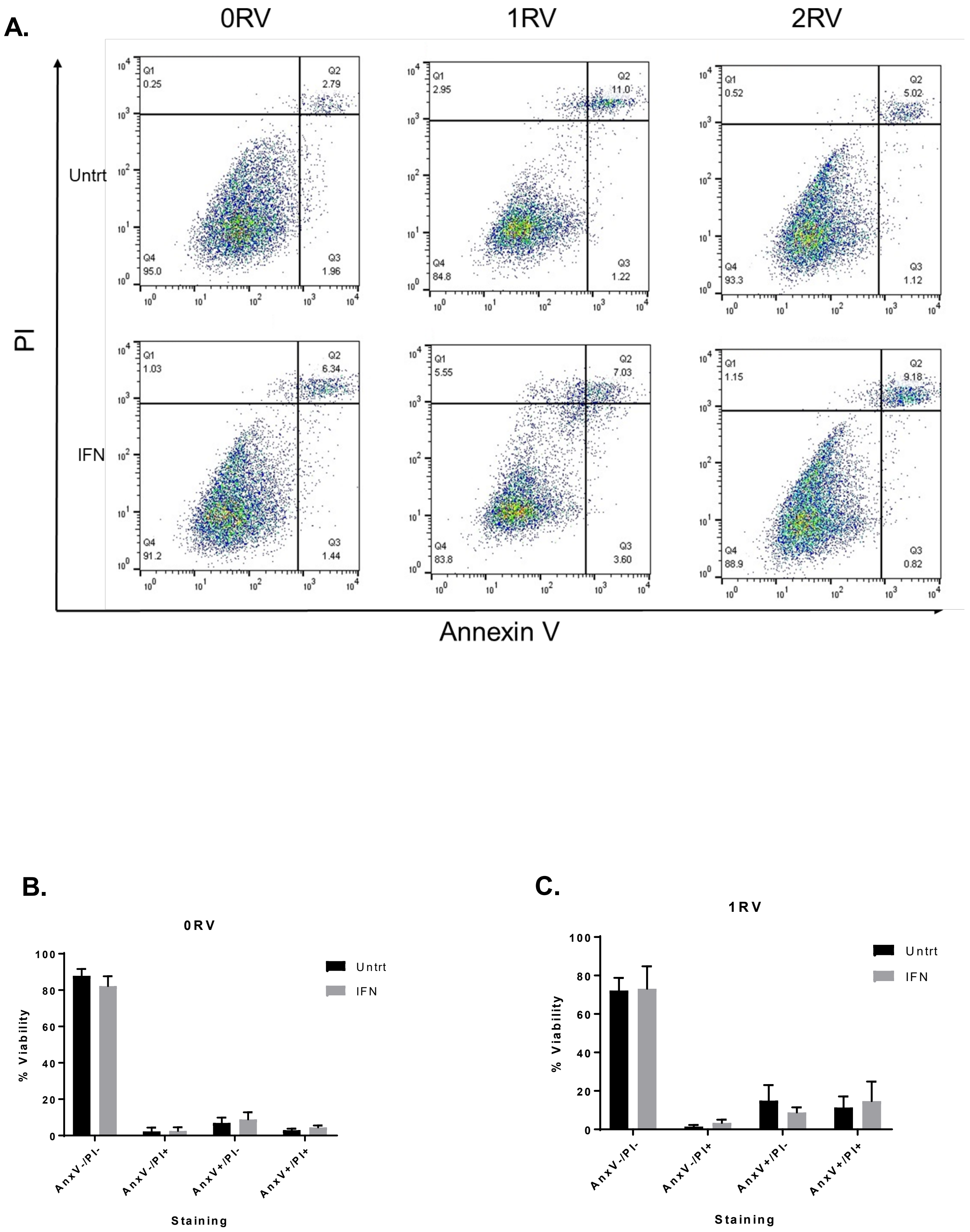

A.
0RV
1RV
2RV
PI
Untrt
IFN
Annexin V
Q1
0.25
Q2
2.79
Q4
95.0
Q3
1.96
Q1
2.95
Q2
11.0
Q4
84.8
Q3
1.22
Q1
0.52
Q2
5.02
Q4
93.3
Q3
1.12
Q1
1.03
Q2
6.34
Q4
91.2
Q3
1.44
Q1
5.55
Q2
7.03
Q4
83.8
Q3
3.60
Q1
1.15
Q2
9.18
Q4
88.9
Q3
0.82
B.
0RV
% Viability
Untrt
IFN
AnxV-/PI-
AnxV-/PI+
AnxV+/PI-
AnxV+/PI+
Staining
C.
1RV
% Viability
Untrt
IFN
AnxV-/PI-
AnxV-/PI+
AnxV+/PI-
AnxV+/PI+
Staining

D.

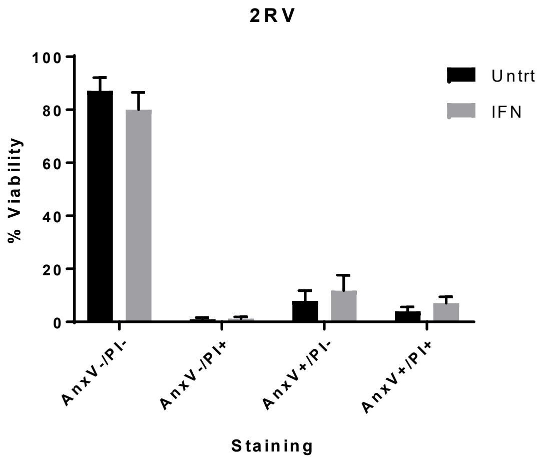

E.

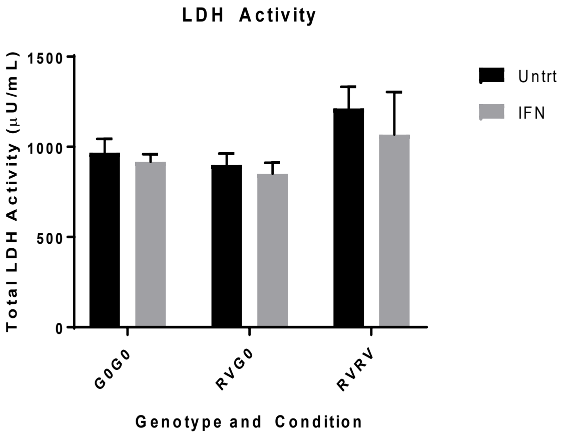

Supplemental Figure Set 3. Live cell metabolic assay by treatment conditions and varied genotypes. **A-E.** Shown on the Y-axis are Bioenergetic health index and on x-axis, treatment condition and genotype for experiments 1-5. Note that conditions employed varied samples (0RV n=4; 1RV n=3; 2RV n=2).

A.

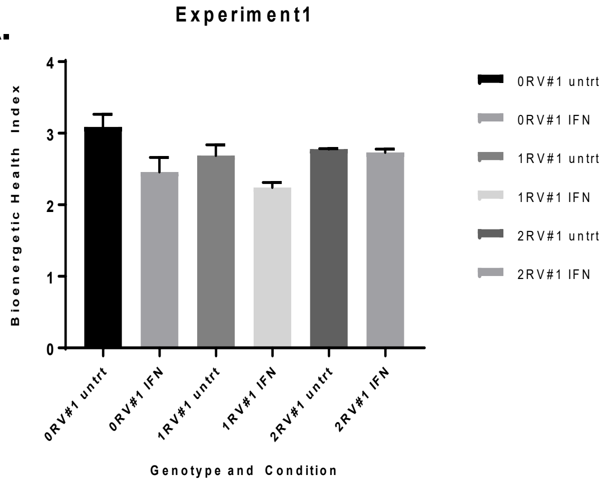

B.

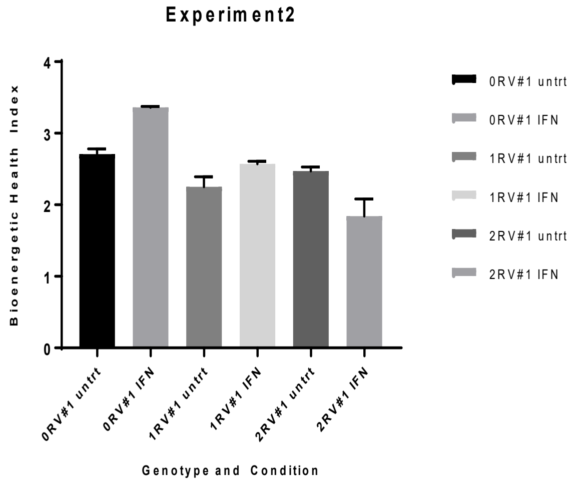

C.

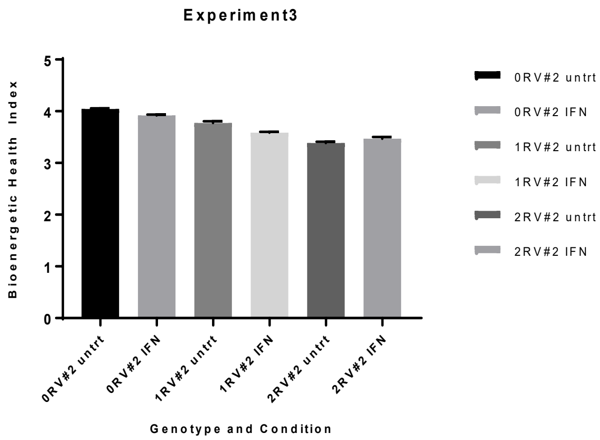

D.

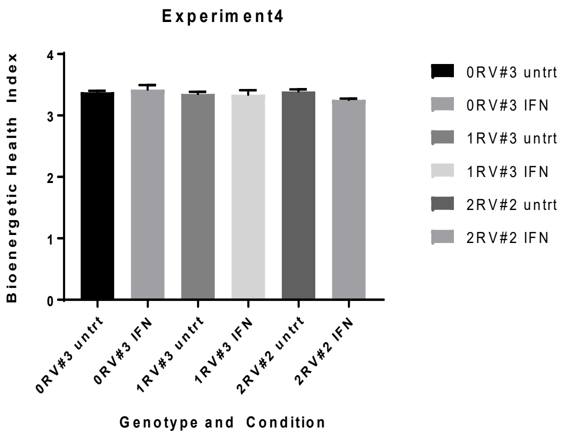

E.

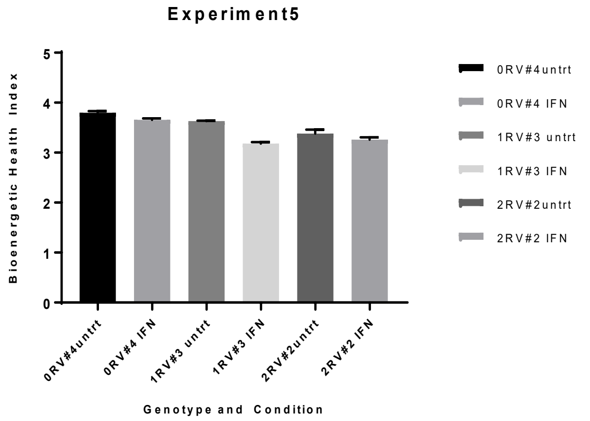

Supplemental Figure Set 4. HUVEC mitochondria by Transmission Electron Microscopy (TEM). Decreased mitochondrial area in IFNɣ- treated HUVECs with 1 RV or 2RV versus IFNɣ treated 0RV HUVECs exposed in parallel. **A.** Representative TEM images of untreated HUVECs. Genotypes shown are as follows: 0 risk variant (0RV left column), 1 risk variant (1RV middle column), and 2 risk variants (2RV right column). **B.** Representative TEM images of HUVECs treated with IFNɣ 1 ng/mL (18 hrs). **C.** TEM images (n=111) were examined across APOL1 genotype and exposure condition. Cytoplasm and organelles were traced using a stoichiometric approach. The proportion of cytoplasm area occupied by mitochondria (both in μm^2) were assessed. There was no statistically significant difference in total mitochondrial area by genotype or treatment condition.

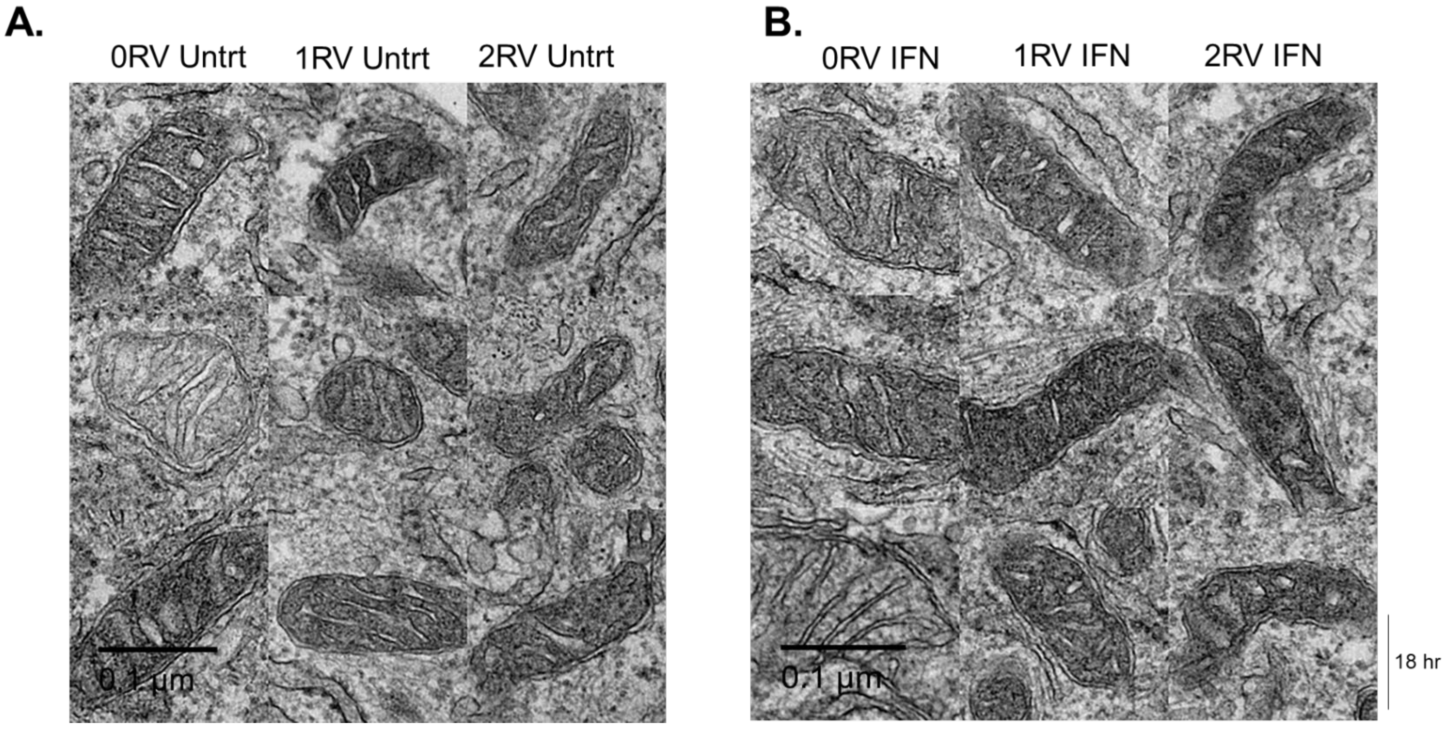

C.

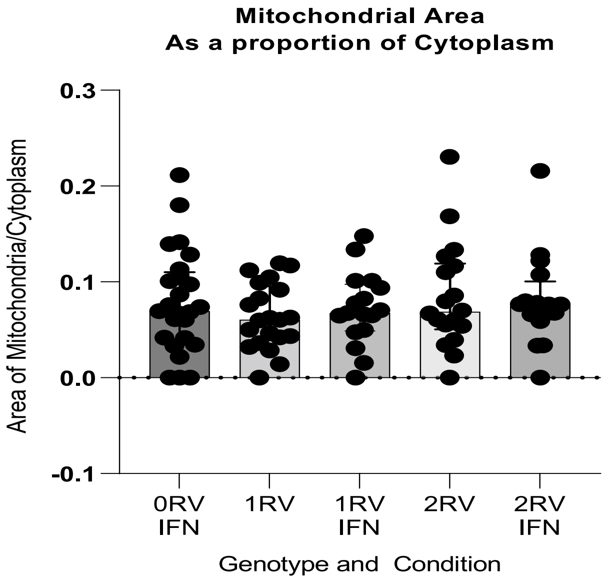

Supplemental Figure 5. APOL1 expression by treatment conditions and experiment. A. Immunoblot of APOL1 and loading control, α tubulin, across genotype—0 risk variant (G0/G0), 1 risk variant (RV/G0), and 2 risk variant (RV/RV). Cells were either left untreated (unt), IFNɣ treated (1 ng/mL, 18 h), or HCQ (25μM, 18h) plus IFNɣ treated. **B.** Dose Response Curve: APOL1 expression in response to titrations of IFN gamma.

A.

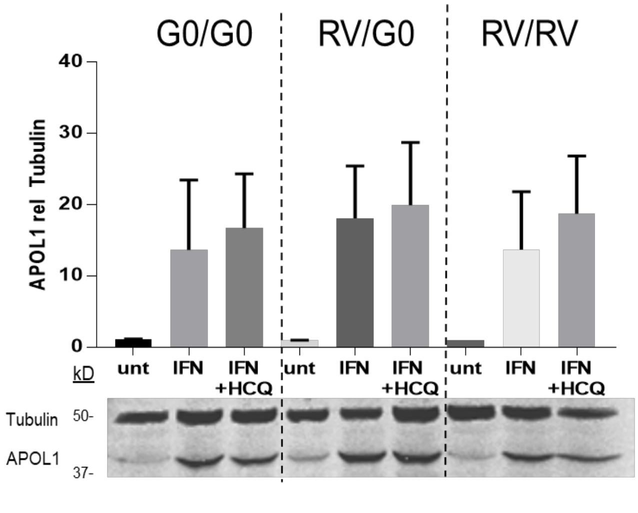

B.

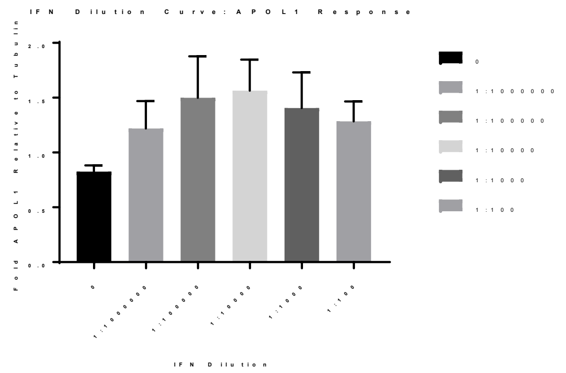

Supplemental Figure Set 6. Lysotracker units (Integrated density) by treatment conditions and experiment. APOL1 risk variants compromise HUVEC lysosome membranes. Lysosome integrative density by experiment (n=4) and sample (G0/G0 n=4; RV/G0 n=3; RV/RV n=2). A representative image is shown

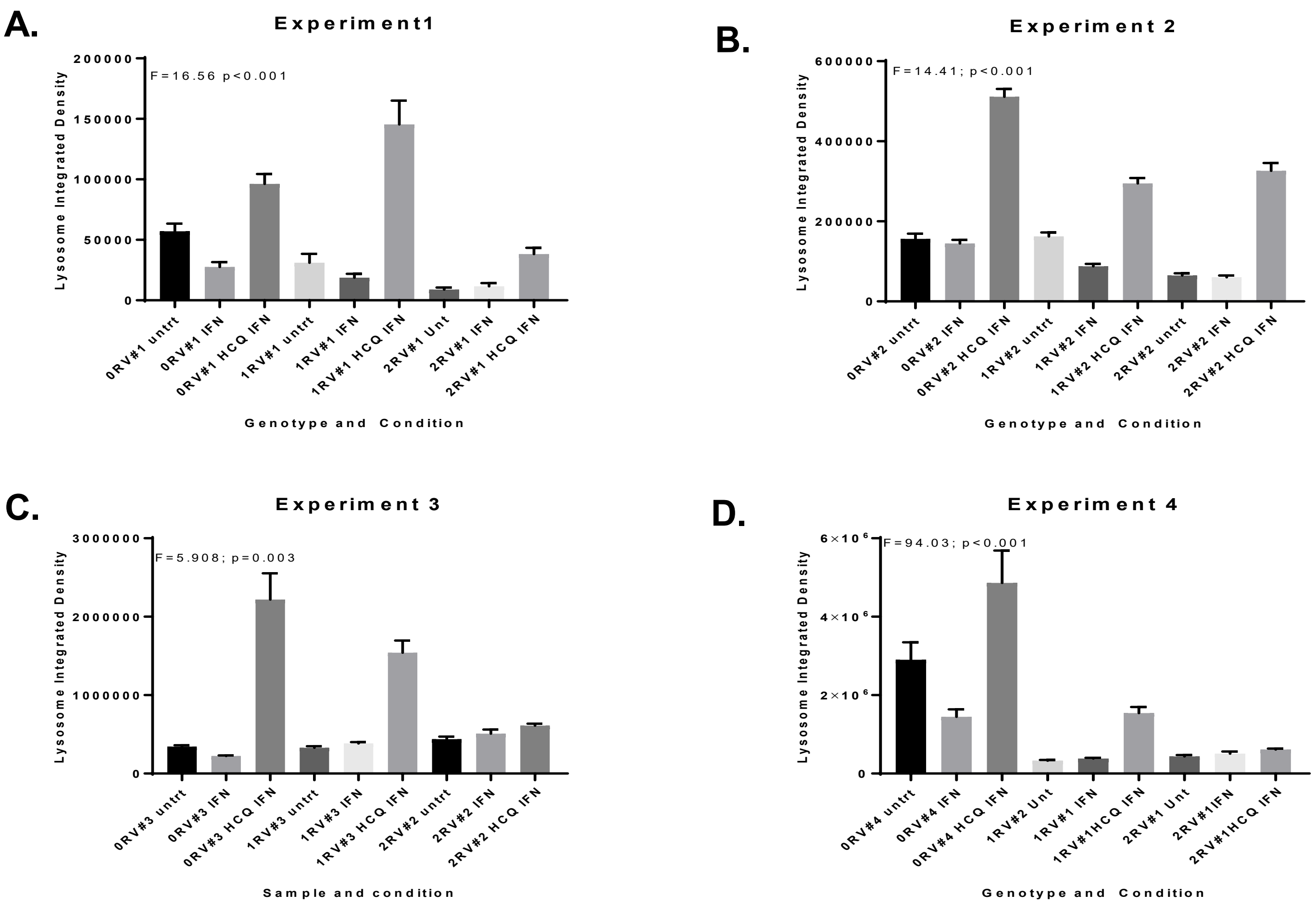
A.
Experiment1
F=16.56 p<0.001
Lysosome Integrated Density
200000
150000
100000
50000
0
0RV#1 untrt
0RV#1 IFN
0RV#1 HCQ IFN
1RV#1 untrt
1RV#1 IFN
1RV#1 HCQ IFN
2RV#1 Unt
2RV#1 IFN
2RV#1 HCQ IFN
Genotype and Condition
B.
Experiment 2
F=14.41; p<0.001
Lysosome Integrated Density
600000
400000
200000
0
0RV#2 untrt
0RV#2 IFN
0RV#2 HCQ IFN
1RV#2 untrt
1RV#2 IFN
1RV#2 HCQ IFN
2RV#2 untrt
2RV#2 IFN
2RV#2 HCQ IFN
Genotype and Condition
C.
Experiment 3
F=5.908; p=0.003
Lysosome Integrated Density
3000000
2000000
1000000
0
0RV#3 untrt
0RV#3 IFN
0RV#3 HCQ IFN
1RV#3 untrt
1RV#3 IFN
1RV#3 HCQ IFN
2RV#2 untrt
2RV#2 IFN
2RV#2 HCQ IFN
Sample and condition
D.
Experiment 4
F=94.03; p<0.001
Lysosome Integrated Density
6×10⁶
4×10⁶
2×10⁶
0
0RV#4 untrt
0RV#4 IFN
0RV#4 HCQ IFN
1RV#2 Unt
1RV#1 IFN
1RV#1HCQ IFN
2RV#1 Unt
2RV#1IFN
2RV#1HCQ IFN
Genotype and Condition

E.

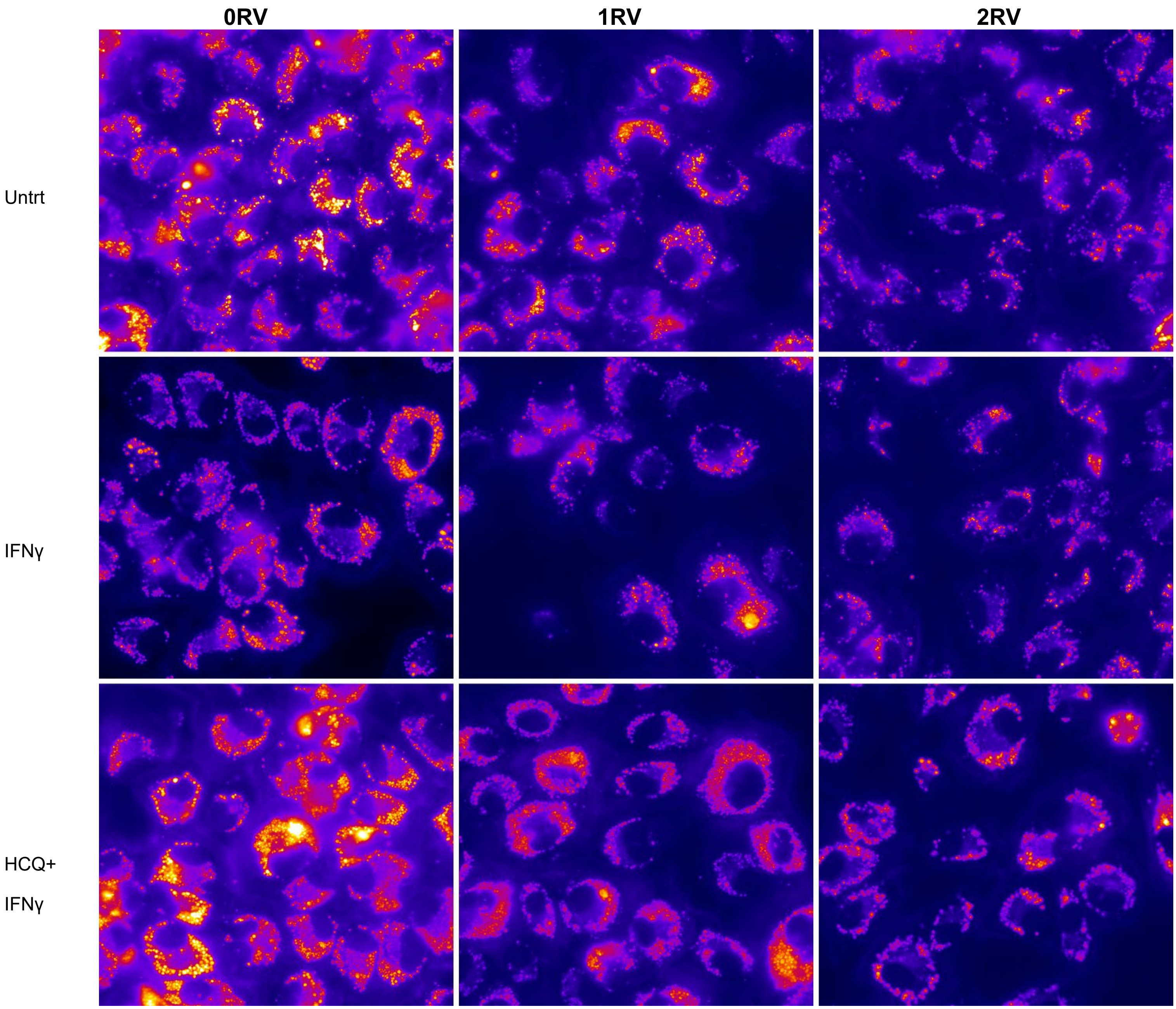
0RV
1RV
2RV
Untrt
IFNγ
HCQ+
IFNγ

Supplemental Figure Set 7. **P62 immunofluorescence units (Autophagosome count) by treatment conditions and experiment. A-D.** APOL1 risk variant carrying HUVECs display autophagic flux deficiencies. Log Autophagosome count by experiment (n=4) and sample (G0/G0 n=4; RV/G0 n=4; RV/RV n=2)

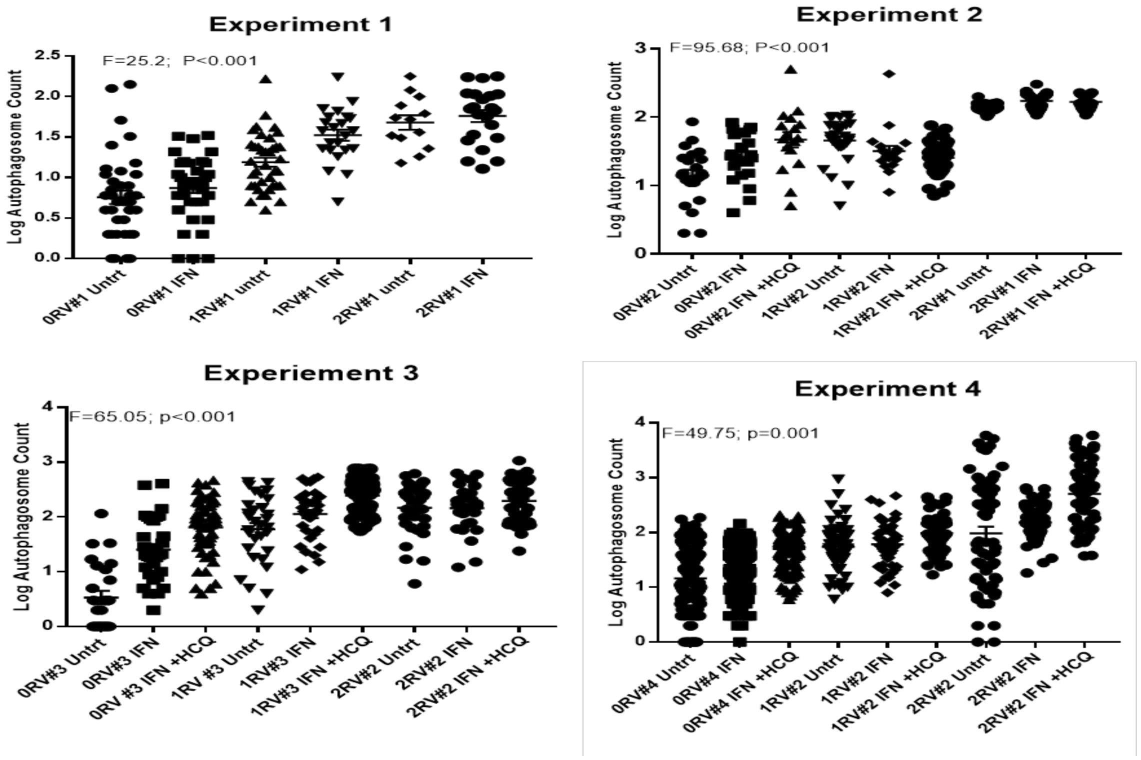

Suppmental Figure Set 8. Autophagosomes were confirmed by transmission electron microscopy (Columns: 0RV left, 1RV middle, 2RV right; Rows: Unt=untreated top, IFNɣ-treated middle, IFNɣ plus HCQ-treated bottom).

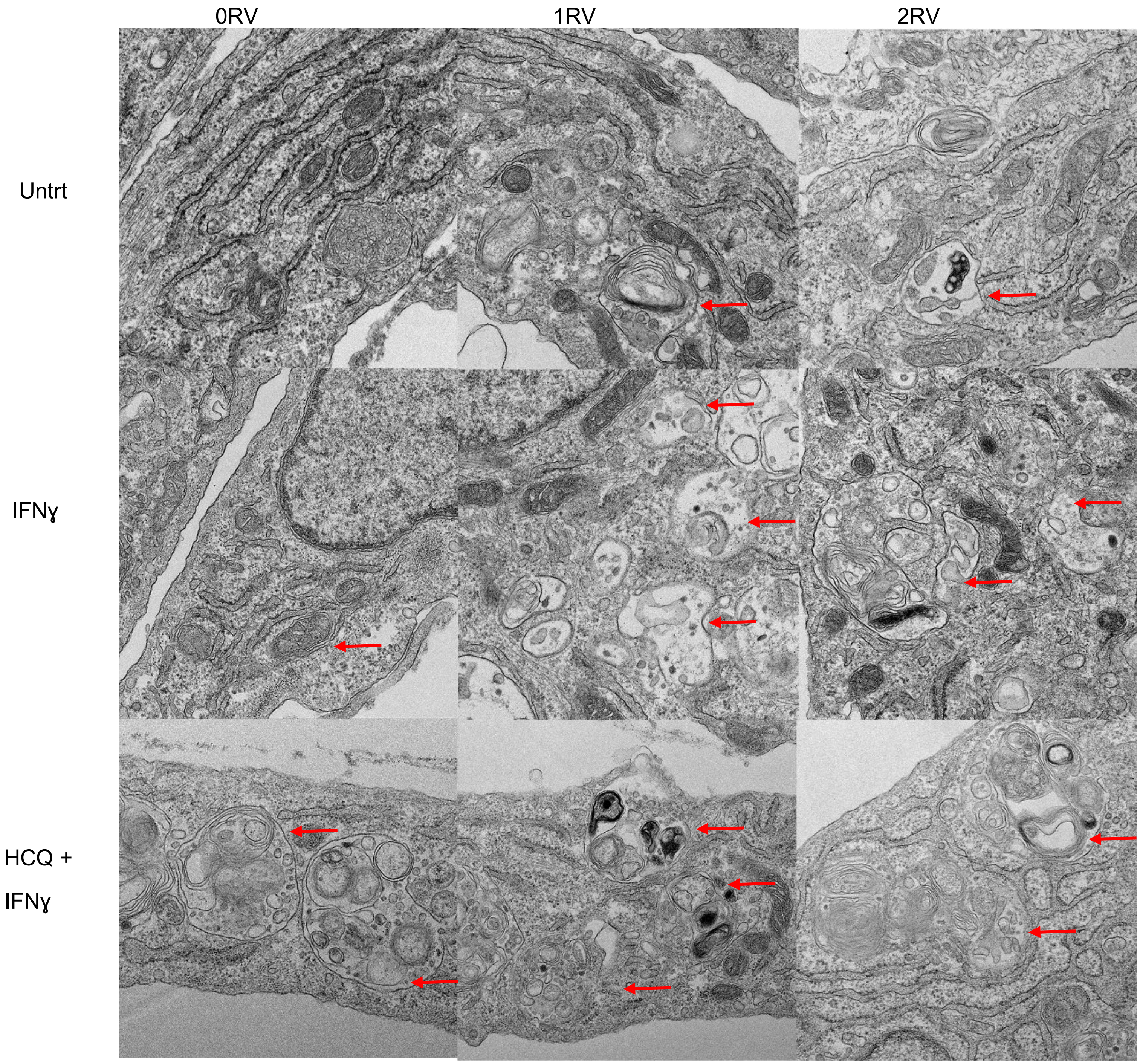

Supplemental Figure 9: Upregulation of LC3 II/ LC3 I ratio, a proxy of autophagosomes, by IFN treated HUVECs with 0RV but not 1RV or 2RV HUVECs. Note the loss of this proxy is associated with autophagic flux inhibition. Immunoblot of HUVEC lysates showing LC3 I and II protein concentration compared to tubulin loading control by genotype and treatment condition. Quantification of HUVEC lysate LC3-II to I ratio (y axis) with anti- tubulin loading control by genotype (left to right) and treatment condition (x axis). Below, a representative Immunoblot is shown.

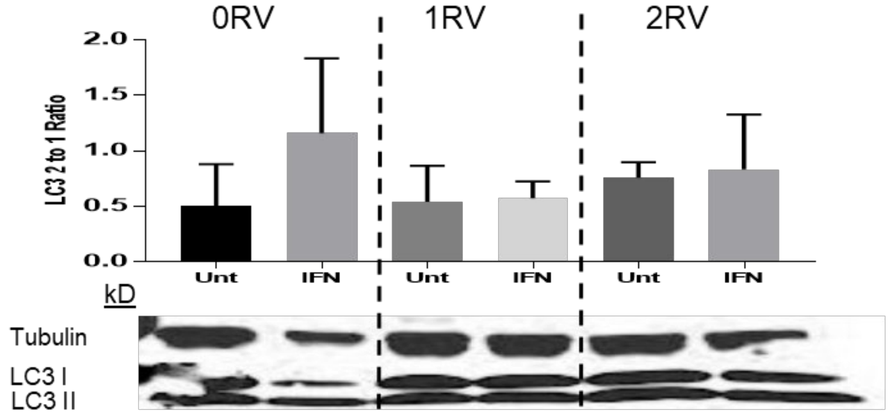

Supplement: Supplementary file 1 [file DataSheet2.PDF]
