# Supplementary material for: APOL1 variant-expressing endothelial cells exhibit autophagic dysfunction and mitochondrial stress
Source: Front Genet. 2022 Sep 27;13:769936. doi: 10.3389/fgene.2022.769936 (PMC9551299; doi:10.3389/fgene.2022.769936)
Supplement: Supplementary file 2 [file DataSheet1.PDF]

Major Resources Tables

Antibodies

Primary Antibodies

Target antigen	Vendor or Source	Catalog #	Working concentration	Lot # (preferred but not required)
CD146	ThermoFisher	50-1469-42	5µL/1mL	
CD31	abcam	Ab9498	1µL/1mL	
α-tubulin	ThermoFisher	236-10501	1:2000	
LC3 A/B rabbit	Cell Signaling	12741	1.3µL/1mL	
SQSTRM1/p62	abcam	ab56416	1µL/1mL	
APOL1	Sigma-Aldrich	HPA018885	1 µL/1mL	
MitoTracker Green	ThermoFisher	M7514	0.08µL/1mL	
LysoTracker Red	ThermoFisher	L7528	0.08µL/1mL	

Secondary Antibodies

Target antigen	Vendor or Source	Catalog #	Working concentration	Lot # (preferred but not required)
**Goat anti-mouse IgG**	abcam	ab216772	1:10000	
**Goat anti-rabbit IgG**	abcam	ab216777	1:10000	
Goat anti-mouse IgG (FITC)	abcam	Ab6785	1:100	
Goat anti-rabbit IgG (TRITC)	abcam	ab6718	1:100	

Cultured Cells

Name	Vendor or Source	Sex (F, M, or unknown)
HUVEC (G0/G0 APOL1)	In house	M=5 F=3
HUVEC (RV/G0 APOL1)	In house	M=1 F=3
HUVEC (RV/RV APOL1)	In house	M=2 F=1
WISH (ATCC CCL-25)	ATCC	unknown
